# Supplementary material for: Sacrum morphology supports taxonomic heterogeneity of “Australopithecus africanus” at Sterkfontein Member 4
Source: Commun Biol. 2021 Mar 17;4:347. doi: 10.1038/s42003-021-01850-7 (PMC7969745; doi:10.1038/s42003-021-01850-7)
Supplement: Supplementary file 1 — Description of Additional Supplementary Files [file 42003_2021_1850_MOESM1_ESM.pdf]

Description of Additional Supplementary Files

File Name: Supplementary Data 1

Description: Morphologika file for the landmark configurations representing 171 hominoid specimens. Note that the number of individuals indicated in the file is 172 because A.L. 288-1 is represented twice (mirrored right and mirrored left side versions). Abbreviations: HS: recent *Homo sapiens*, adults; JUV: recent *Homo sapiens*, juveniles; GB: *Gorilla beringei*; GG: *Gorilla gorilla*; PP: *Pan paniscus*; PT: *Pan troglodytes*; PA: *Pongo abelii*; PPYG: *Pongo pygmaeus*; m: male; f: female

File Name: Supplementary Data 2

Description: Data underlying the plots in Fig. 2 and Fig. 3, and the column distributions in Fig. 4

File Name: Supplementary Movie 1

Description: GIF animation of the 3D PCA plot for all individuals (blue = modern human adults; sky blue = modern human subadults; medium orchid = *Pan*; green = *Gorilla*; orange = *Pongo*) after Generalized Procrustes Analysis, in shape space.

File Name: Supplementary Movie 2

Description: GIF animation of the 3D PCA plot for modern humans (blue = males; sky blue = females), Sts 14 and StW 431 after Generalized Procrustes Analysis, in shape space.
