# Supplementary figures and images for: Sacrum morphology supports taxonomic heterogeneity of “Australopithecus africanus” at Sterkfontein Member 4
Source: Commun Biol. 2021 Mar 17;4:347. doi: 10.1038/s42003-021-01850-7 (PMC7969745; doi:10.1038/s42003-021-01850-7)

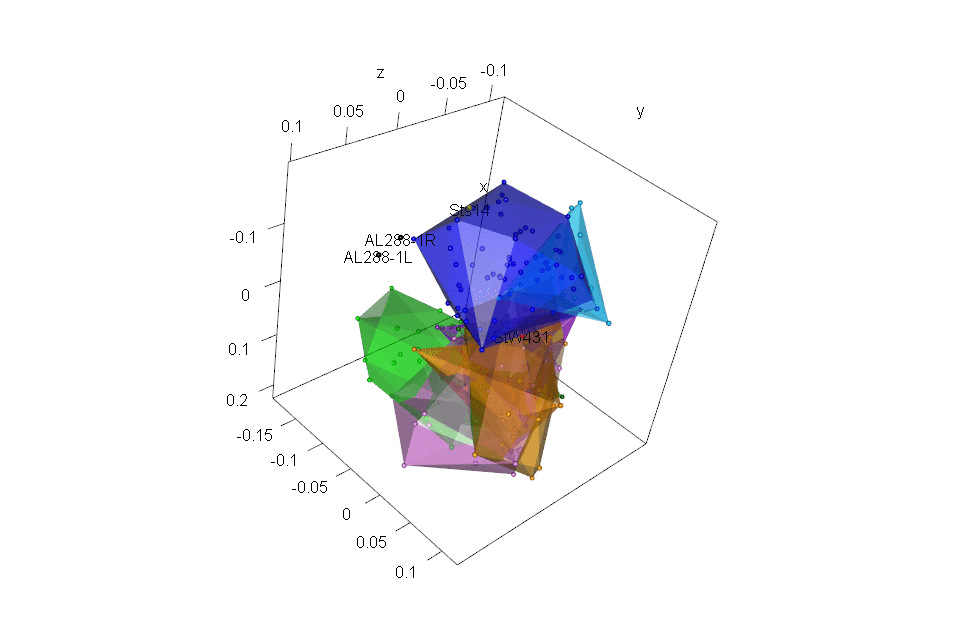

Supplement: Supplementary file 2 — Supplementary Movie 1 [file 42003_2021_1850_MOESM2_ESM.gif]

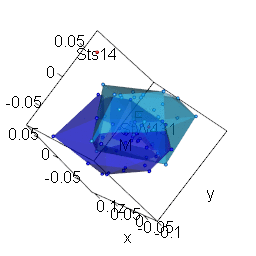

Supplement: Supplementary file 3 — Supplementary Movie 2 [file 42003_2021_1850_MOESM3_ESM.gif]
