# Supplementary material for: Sacrum morphology supports taxonomic heterogeneity of “Australopithecus africanus” at Sterkfontein Member 4
Source: Commun Biol. 2021 Mar 17;4:347. doi: 10.1038/s42003-021-01850-7 (PMC7969745; doi:10.1038/s42003-021-01850-7)
Supplement: Supplementary file 4 — Supplementary Information [file 42003_2021_1850_MOESM4_ESM.pdf]

Sacrum morphology supports taxonomic heterogeneity of "*Australopithecus africanus*" at Sterkfontein Member 4

Cinzia Fornai[1,2]*, Viktoria A. Krenn[1,2], Philipp Mitteroecker[3], Nicole M. Webb[1,4], Martin Haeusler[1]

[1]Institute of Evolutionary Medicine, University of Zurich.

[2]Department of Evolutionary Anthropology, University of Vienna.

[3]Department of Theoretical Biology, University of Vienna.

[4]Senckenberg Research Institute and Natural History Museum Frankfurt.

*Corresponding author: cinzia.fornai@univie.ac.at

Supplementary Information

Supplementary Note 1: Quadratic Discriminant Analysis (QDA)

Since StW 431 plotted in an intermediate position between *Homo* and *Pongo* along PC1 and PC2 of shape space, a QDA (see, for example, ref. 56, section 3.6) was carried out to assess the likelihood that StW 431 could be morphologically allied with either *Homo* or *Pongo*. The analysis was performed in the R software environment [59] using a script prepared and supplied by Fred L. Bookstein. The models were built considering only the modern human adults and leaving out StW 431. The p ~0.05 threshold for the hypothesis that StW 431 belongs to *Homo* is 6.83, and that for the hypothesis that StW 431 belongs to *Pongo* it is 0.146. Intermediate values between 0.146 and 6.83 reflect equivocal results. The likelihood ratio (LR) for the hypothesis that StW 431 belongs to *Homo* is 33.90 for PC1-PC2 and 38.44 for PC2-PC3, thereby indicating clear morphological affinity to *Homo*.

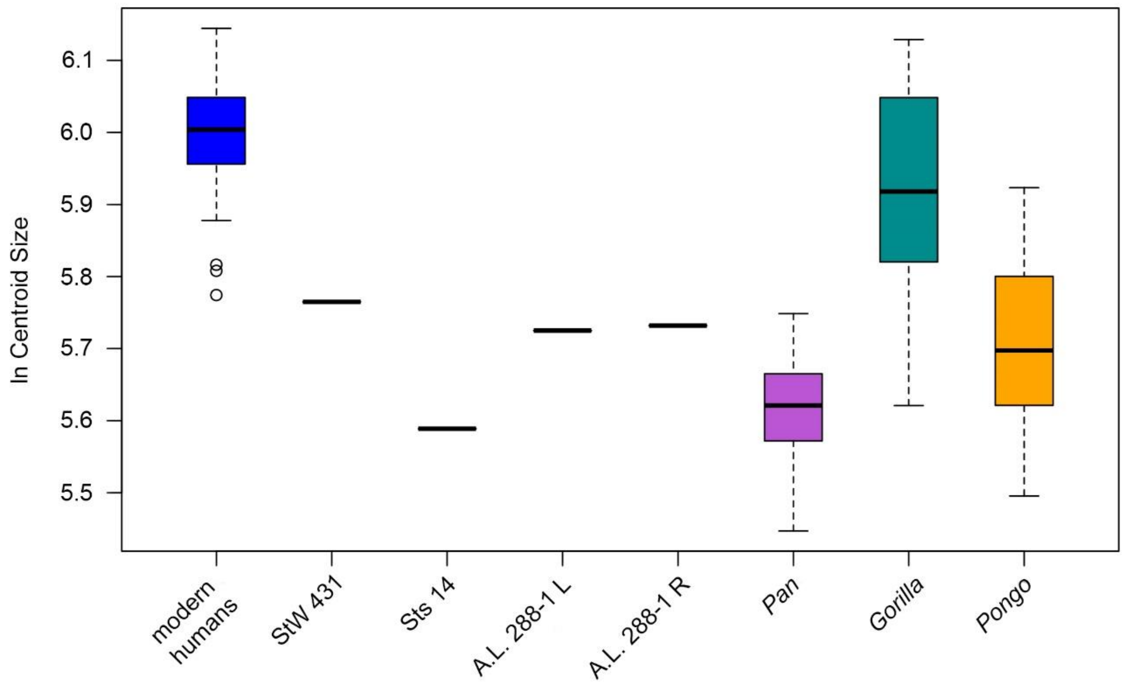

Supplementary Figure 1: Boxplot of ln centroid size. Sts 14 and A.L. 288-1 were smaller than any recent modern humans considered, while StW 431 was at the lower end of the modern human distribution. The ln centroid sizes of the *Australopithecus* sacra were in the range of *Pan* and *Pongo*, and of the smallest *Gorilla* specimens. Sts 14 was smaller than any modern human specimen, including the subadults, and fell within the range of variation of *Pan* and *Pongo*, while StW 431 was at the lower end of the distribution of *Homo*. The regression analysis showed that the association of size on shape accounted for 12.3% of total shape variance in the full sample but for only 3.3% of total shape variance in the hominin subsample.

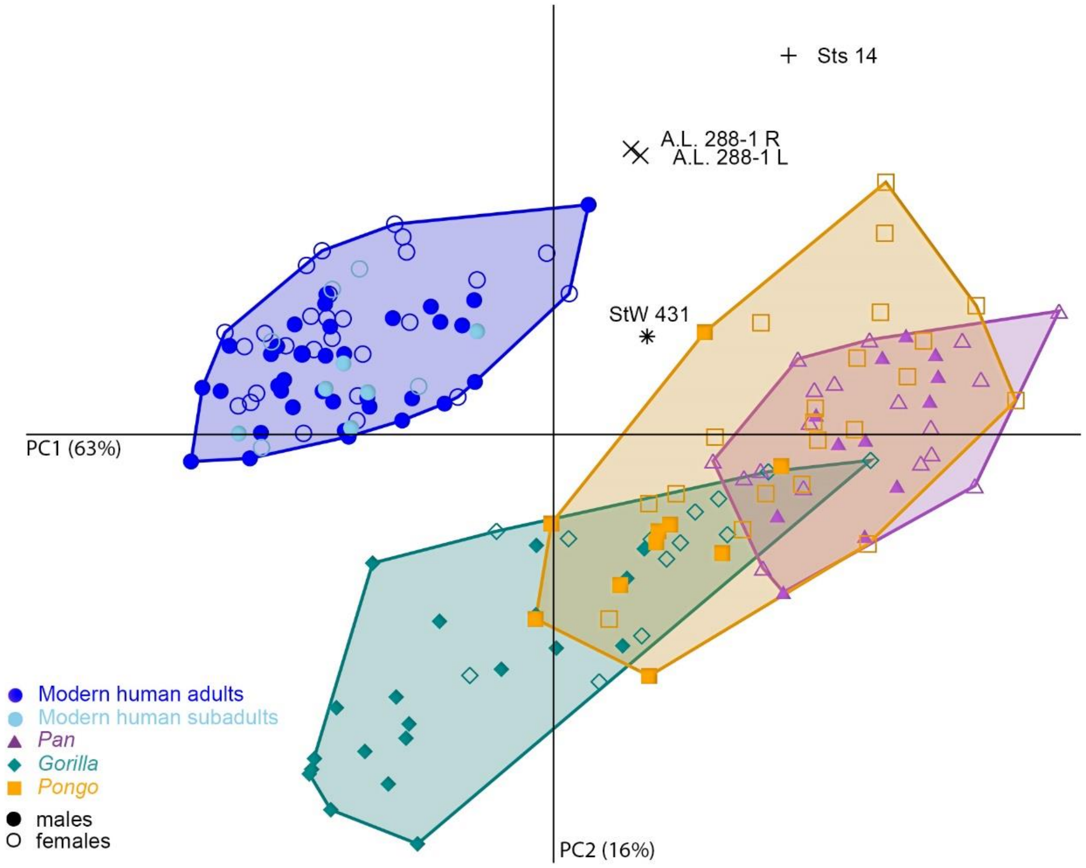

Supplementary Figure 2: PCA plot in form space after Generalized Procrustes Analysis for the sample including both modern humans and great apes. In this plot, modern humans (blue circles = adults; sky-blue circles = subadults) separate completely from great apes (medium-orchid triangles = *Pan*; dark-cyan diamonds = *Gorilla*; orange squares = *Pongo*). In this analysis, the male gorillas and orangutans (closed symbols = males, open symbols = females) tended to separate from the rest of the great apes for their large size. There was still a complete overlap of male and female individuals in modern humans and *Pan*, whereas a dimorphic trend was observed both in *Pongo* and especially *Gorilla*. The *Australopithecus* individuals were distinct from the rest of the sample.

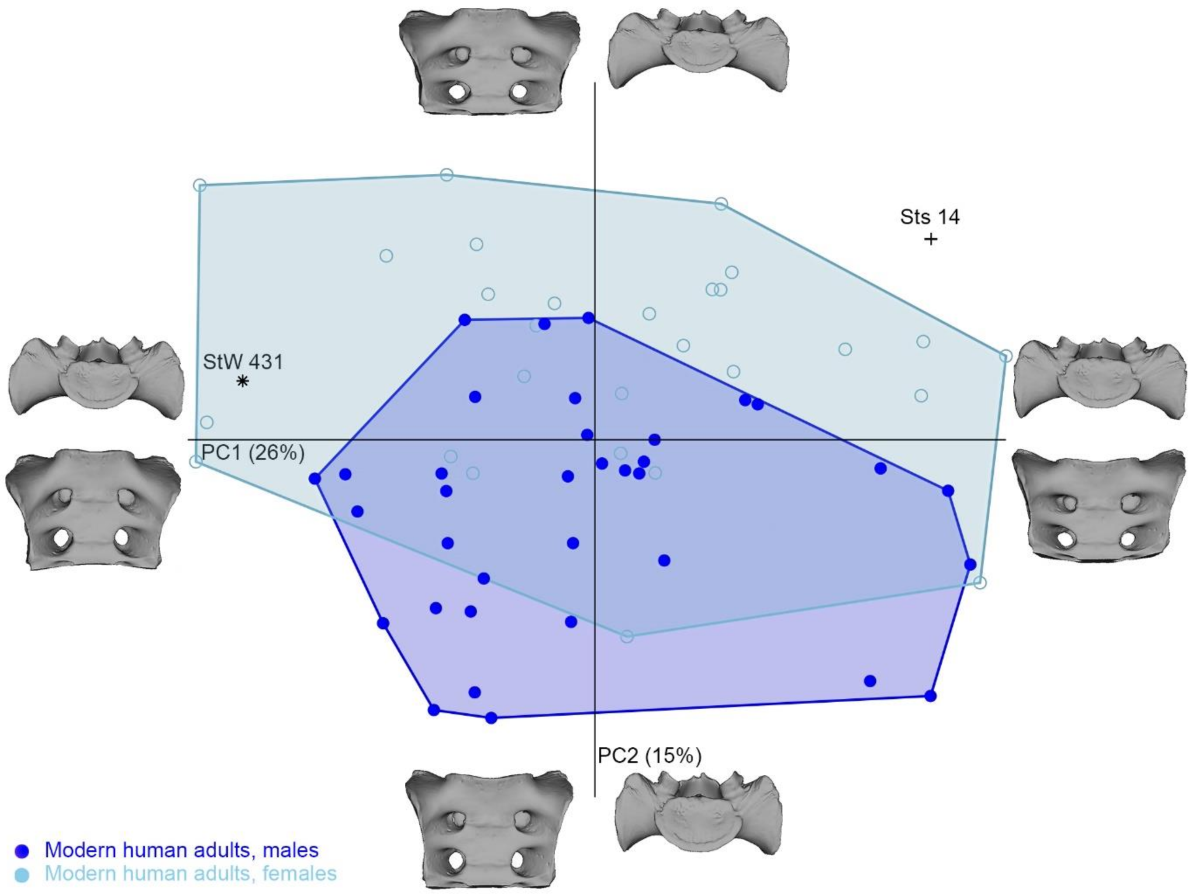

Supplementary Figure 3: PCA plot in shape space after GPA for the modern human adults as well as Sts 14 and StW 431. In the PC1-PC2 plot, male (blue) and female (sky blue) modern humans overlapped despite a clear difference in mean shape. Sts 14 and StW 431 diverged largely along PC1, which reflects the different cranio-caudal orientation of the wings. When the Procrustes distances were calculated based on this PC, only 1.0% of the pairwise comparisons between all modern humans and 1.1% between the male-female pairs were higher than the distances between Sts 14 and StW 431. Sex-related variation, corresponding to the relative width of the wings with respect to the sacral body, occurred in PC2, along which Sts 14 and StW 431 primarily diverged.

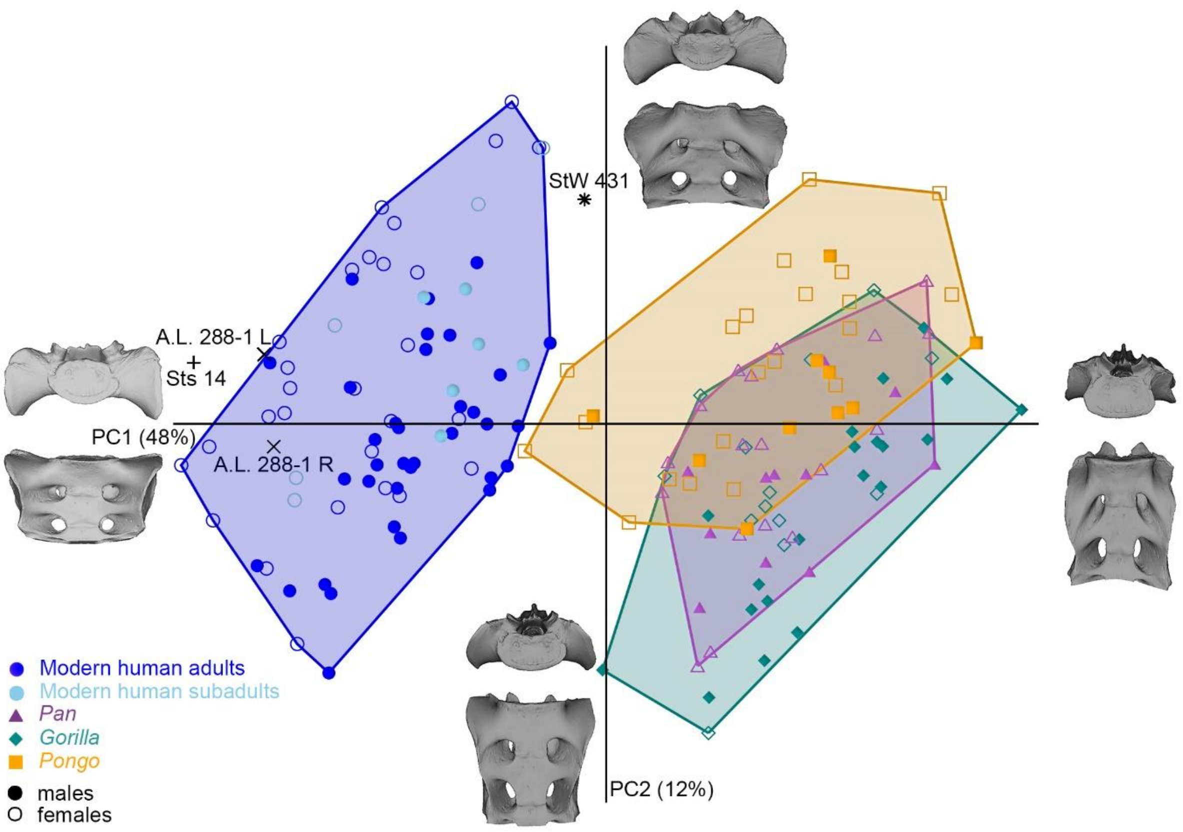

Supplementary Figure 4: PCA plot of the shape coordinates after Procrustes superimposition based on a subset of 13 landmarks on the body of the first sacral vertebra. This analysis emphasizes the relative dimensions of the wings in relation to the sacral body. Sts 14 plotted at an extreme of the modern human distribution, and StW 431 plotted on the other side, between modern humans (blue dot = adults; sky-blue dot = subadults) and great apes (medium-orchid triangles = *Pan*; dark-cyan diamonds = *Gorilla*; orange squares = *Pongo*). These outcomes highlight the large differences between Sts 14 and StW 431 in terms of width-to-height proportions. Modern humans were distinct from the great apes along PC1, whereas *Pan, Gorilla,* and *Pongo* overlapped extensively. Closed symbols = males, open symbols = females.

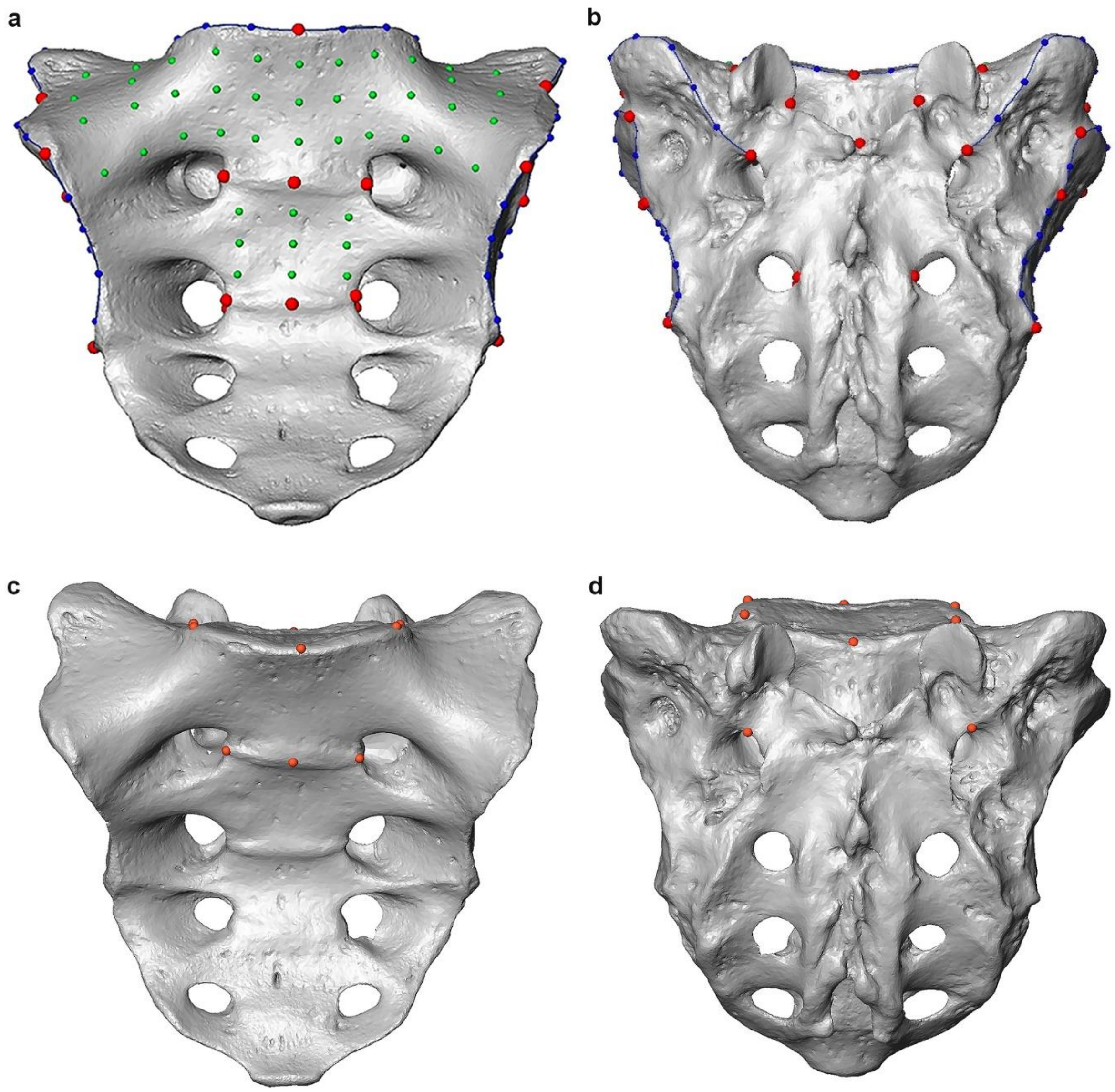

Supplementary Figure 5: The landmark configuration used in this study. a Anterior and **b** posterior views of a modern human sacrum. Landmarks: red points; curve semilandmarks: blue points on blue lines; surface semilandmarks: green points. **c** anterior and **d** posterior views showing the 13 landmarks (orange) used for the Procrustes fit based on a subset of landmarks.
