# Supplementary material for: Sacrum morphology supports taxonomic heterogeneity of “Australopithecus africanus” at Sterkfontein Member 4
Source: Commun Biol. 2021 Mar 17;4:347. doi: 10.1038/s42003-021-01850-7 (PMC7969745; doi:10.1038/s42003-021-01850-7)
Supplement: Supplementary file 7 — Reporting Summary [file 42003_2021_1850_MOESM7_ESM.docx]

1


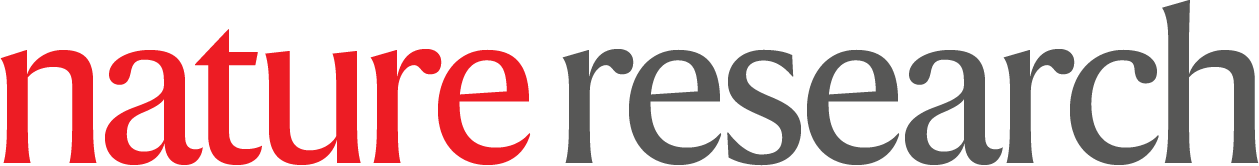
Corresponding author(s): COMMSBIO-20-2644-T

nature research | reporting summary

Last updated by author(s): Jan 28, 2021

Reporting Summary

Nature Research wishes to improve the reproducibility of the work that we publish. This form provides structure for consistency and transparency in reporting. For further information on Nature Research policies, see our Editorial Policies and the Editorial Policy Checklist.

Please do not complete any field with "not applicable" or n/a. Refer to the help text for what text to use if an item is not relevant to your study. For final submission: please carefully check your responses for accuracy; you will not be able to make changes later.

## Statistics

For all statistical analyses, confirm that the following items are present in the figure legend, table legend, main text, or Methods section. n/a Confirmed


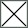

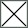

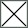

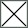

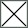

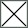


The exact sample size (*n*) for each experimental group/condition, given as a discrete number and unit of measurement

A statement on whether measurements were taken from distinct samples or whether the same sample was measured repeatedly The statistical test(s) used AND whether they are one- or two-sided

*Only common tests should be described solely by name; describe more complex techniques in the Methods section.*

A description of all covariates tested

A description of any assumptions or corrections, such as tests of normality and adjustment for multiple comparisons

A full description of the statistical parameters including central tendency (e.g. means) or other basic estimates (e.g. regression coefficient) AND variation (e.g. standard deviation) or associated estimates of uncertainty (e.g. confidence intervals)

For null hypothesis testing, the test statistic (e.g. *F*, *t*, *r*) with confidence intervals, effect sizes, degrees of freedom and *P* value noted

*Give P values as exact values whenever suitable.*

For Bayesian analysis, information on the choice of priors and Markov chain Monte Carlo settings

For hierarchical and complex designs, identification of the appropriate level for tests and full reporting of outcomes Estimates of effect sizes (e.g. Cohen's *d*, Pearson's *r*), indicating how they were calculated

*Our web collection on statistics for biologists contains articles on many of the points above.*

## Software and code

Policy information about availability of computer code Data collection

Amira (https://www.thermofisher.com) for 3D image data segmentation. Viewbox [4 (http://www.dhal.com/viewboxindex.htm)](http://www.dhal.com/viewboxindex.htm)) for landmark collection

Data analysis

Evan Toolbox [(https://www.evan-society.org/)](http://www.evan-society.org/)) for standard geometric morphometrics. R software [(https://www.r-project.org/)](http://www.r-project.org/)) for geometric morphometrics after Procrustes fit on a subset of landmarks; computation of percentages of Procrustes distances and group mean differences. All analyses were rechecked in Mathematica (https://www.wolfram.com)

For manuscripts utilizing custom algorithms or software that are central to the research but not yet described in published literature, software must be made available to editors and reviewers. We strongly encourage code deposition in a community repository (e.g. GitHub). See the Nature Research guidelines for submitting code & software for further information.

## Data

Policy information about availability of data

All manuscripts must include a data availability statement. This statement should provide the following information, where applicable:

*April 2020*

- Accession codes, unique identifiers, or web links for publicly available datasets
- A list of figures that have associated raw data
- A description of any restrictions on data availability

The authors declare that the data supporting the findings of this study are available within the paper and its supplementary information files. In particular, the landmark data are available as supplementary data file.

2

*April 2020*

# Field-specific reporting

nature research | reporting summary

Please select the one below that is the best fit for your research. If you are not sure, read the appropriate sections before making your selection.

Life sciences Behavioural & social sciences Ecological, evolutionary & environmental sciences

For a reference copy of the document with all sections, see nature.com/documents/nr-reporting-summary-flat.pdf

# Life sciences study design

All studies must disclose on these points even when the disclosure is negative. Sample size

The sample was chosen based on specimen availability in the fossil and osteological collections

Data exclusions

Specimens lacking regions of interest, showing pathologies or patent asymmetry were excluded

Replication

Landmarks were collected and carefully double-checked by two experienced observers. An intra- and inter-observer error assessment was performed by C.F. and V.A.K. that confirmed high accuracy and precision of the landmark configuration (highest Procrustes distance between all repeats was lower than 2700 Procrustes Distances out of 2701 possible univocal combinations derived from sample of 74 human

sacra).’ [(((74x74)-74)/2)=2701]. Analyses were performed independently by two of us.

Randomization

Randomization does not apply here because group assignments were determined by the species affiliation

Blinding

Blinding did not apply here, but the data were collected and carefully double-checked by two observers

# Reporting for specific materials, systems and methods

We require information from authors about some types of materials, experimental systems and methods used in many studies. Here, indicate whether each material, system or method listed is relevant to your study. If you are not sure if a list item applies to your research, read the appropriate section before selecting a response.

### Materials & experimental systems Methods


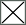

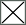

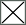

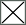

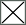

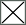


n/a Involved in the study Antibodies Eukaryotic cell lines

Palaeontology and archaeology Animals and other organisms Human research participants Clinical data

Dual use research of concern


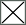

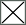

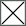


n/a Involved in the study

ChIP-seq

Flow cytometry

MRI-based neuroimaging

Palaeontology and Archaeology

Sts 14q: Ditsong National Museum of Natural History, Pretoria. StW 431h: Evolutionary Studies Institute, University of the Witwatersrand, Johannesburg, South Africa. A.L. 288-1an: National Museum of Ethiopia, Addis Ababa, Ethiopia. Modern human adults: Anthropological Institute and Museum, University of Zurich, Switzerland; Department of Anthropology and Narrenturm, Natural History Museum of Vienna, Austria; Department of Evolutionary Anthropology, University of Vienna, Austria; Institute of Evolutionary Medicine, University of Zurich, Switzerland; Laboratory of Prehistoric Archaeology and Anthropology, University of Geneva, Switzerland; Museum of Natural History, University of Florence, Italy; Smithsonian National Museum of Natural History, Washington, USA. Modern human subadults: Hospital Timone, Marseille, France. Gorilla beringei: Anthropological Institute and Museum, University of Zurich, Switzerland; Laboratory of Prehistoric Archaeology and Anthropology, University of Geneva, Switzerland; Department of Zoology, Natural History Museum of Vienna, Austria; Zoological Museum, University of Zurich, Switzerland. Gorilla gorilla: Anthropological Institute and Museum, University of Zurich, Switzerland; Laboratory of Prehistoric Archaeology and Anthropology, University of Geneva, Switzerland; Department of Zoology, Natural History Museum of Vienna, Austria; Zoological Museum, University of Zurich, Switzerland; Digital Morphology Museum, KUPRI, Kyoto University, Japan. Pan paniscus: Royal Museum for Central Africa, Tervuren, Belgium. Pan troglodytes: Anthropological Institute and Museum, University of Zurich, Switzerland; Laboratory of Prehistoric Archaeology and Anthropology, University of Geneva, Switzerland; Department of Zoology, Natural History Museum of Vienna, Austria; Zoological Museum, University of Zurich, Switzerland; Digital Morphology Museum, KUPRI, Kyoto University, Japan; Museum of Primatology, University of California, San Diego; Natural History Museum Basel, Switzerland. Pongo abelii: Anthropological Institute and Museum, University of Zurich, Switzerland; Laboratory of Prehistoric Archaeology and Anthropology, University of Geneva, Switzerland. Pongo pygmaeus: Anthropological Institute and Museum, University of Zurich, Switzerland; Laboratory of Prehistoric Archaeology and Anthropology, University of Geneva, Switzerland; Department of Zoology, Natural History Museum of Vienna, Austria; Digital Morphology Museum, KUPRI, Kyoto University, Japan; Museum of Primatology, University of California, San Diego.

Specimen provenance

3

Specimen deposition

All specimens are located at their original repositories

nature research | reporting summary

Dating methods

No new dates were provided

Tick this box to confirm that the raw and calibrated dates are available in the paper or in Supplementary Information.

Ethics oversight

No ethical approval was applied since the project involved exclusively fossil and osteological specimens and used no destructive approaches. We referred to the Code of Ethics for Research on Human Remains of the Institute of Evolutionary Medicine, University of Zurich, https://www.iem.uzh.ch/dam/jcr:ffffffff-d4ad-95ae-ffff-ffff95c95f9c/Code_of_Ethics_IEM_2014.pdf

Note that full information on the approval of the study protocol must also be provided in the manuscript.


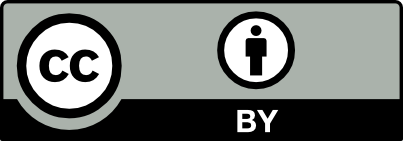
This checklist template is licensed under a Creative Commons Attribution 4.0 International License, which permits use, sharing, adaptation, distribution and reproduction in any medium or format, as long as you give appropriate credit to the original author(s) and the source, provide a link to the Creative Commons license, and indicate if changes were made. The images or other third party material in this article are included in the article's Creative Commons license, unless indicated otherwise in a credit line to the material. If material is not included in the article's Creative Commons license and your intended use is not permitted by statutory regulation or exceeds the permitted use, you will need to obtain permission directly from the copyright holder. To view a copy of this license, visit <http://creativecommons.org/licenses/by/4.0/>

*April 2020*
